# Ellagic Acid Attenuates BLM-Induced Pulmonary Fibrosis via Inhibiting Wnt Signaling Pathway

**DOI:** 10.3389/fphar.2021.639574

**Published:** 2021-04-12

**Authors:** Xiaohe Li, Kai Huang, Xiaowei Liu, Hao Ruan, Ling Ma, Jingjing Liang, Yunyao Cui, Yanhua Wang, Shuyang Wu, Hailong Li, Yuli Wei, Zeping Li, Jingjing Gao, Bo Yang, Xiaoping Li, Guang Yang, Honggang Zhou, Cheng Yang

**Affiliations:** ^1^ State Key Laboratory of Medicinal Chemical Biology, College of Pharmacy and Tianjin Key Laboratory of Molecular Drug Research, Nankai University, Tianjin, China; ^2^ Tianjin Key Laboratory of Molecular Drug Research, Tianjin International Joint Academy of Biomedicine, Tianjin, China; ^3^ Department of Thoracic Surgery, Tianjin First Central Hospital, Tianjin, China

**Keywords:** pulmonary fibrosis, ellagic acid, myofibroblasts, autophagy, apoptosis, wnt

## Abstract

Idiopathic pulmonary fibrosis is a progressive lung disease with high mortality and limited therapy that is characterized by epithelial cell damage and fibroblast activation. Ellagic acid is a natural polyphenol compound widely found in fruits and nuts that has multiple pharmacological activities. In this study, we explored the potential effects and mechanisms of Ellagic acid on pulmonary fibrosis *in vivo* and *in vitro*. *In vivo* studies showed that Ellagic acid significantly alleviated bleomycin (BLM)-induced pulmonary fibrosis in mice. *In vitro* experiments indicated that Ellagic acid could suppress Wnt signaling and attenuate Wnt3a-induced myofibroblast activation and the phosphorylation of Erk2 and Akt. Further studies showed that Ellagic acid could induce autophagy formation in myofibroblasts mainly by suppressing mTOR signaling and promoting apoptosis of myofibroblasts. *In vivo* experiments revealed that Ellagic acid significantly inhibited myofibroblast activation and promoted autophagy formation. Taken together, our results show that Ellagic acid effectively attenuates BLM-induced pulmonary fibrosis in mice by suppressing myofibroblast activation and promoting autophagy and apoptosis of myofibroblasts by inhibiting the Wnt signaling pathway.

## Introduction

Idiopathic pulmonary fibrosis is a progressive, fatal and age-associated disease, and the average survival time of IPF patients is only 2–5 years after diagnosis (Directors and Committie, 2000; Thannickal et al., 2004; Mora et al., 2017). In Europe and North America, the incidence range is 3–9 cases per 100,000 per year (Hutchinson et al., 2015). Although the pathogenesis is not completely understood, many researchers believe that myofibroblast activation, autophagy and apoptosis are essential factors in fibrotic progression (Wynn, 2011).

The Wnt/β-catenin signal transduction pathway is essential for adult life and could control a myriad of biological phenomena throughout development (Clevers and Nusse, 2012). Wnt signaling also plays an essential role in fibrotic disease, and inhibiting this pathway could suppress fibrotic progression (Distler et al., 2019). In lung epithelial cells and fibroblasts, β-catenin is overexpressed under fibrotic conditions (Baarsma and Konigshoff, 2017). Many studies have revealed that Wnt ligands induce fibroblast activation and collagen synthesis and that blocking Wnt/β-catenin signaling attenuates BLM-induced pulmonary fibrosis (Konigshoff et al., 2008). Overactive fibroblasts can produce abundant extracellular matrix (ECM) proteins and inducing apoptosis of overactive fibroblasts is regarded as an effective method to alleviate fibrotic diseases (Hosseinzadeh et al., 2018). In breast cancer cells, blockade of Wnt signaling significantly induces cellular apoptosis (Bilir et al., 2013). Hence, inhibiting fibroblast activation and promoting myofibroblast apoptosis could attenuate pulmonary fibrosis by suppressing Wnt/β-catenin signaling.

There is an intimate relationship between autophagy and pulmonary fibrosis. The three main types of autophagy are chaperone-mediated autophagy, microautophagy and macroautophagy (Rubinsztein et al., 2007). Here, we focus on macroautophagy (hereafter referred to as autophagy), and the autophagic pathway is the catabolic mechanism for cytoplasmic organelles and degrading long-lived cellular proteins (Levine and Kroemer, 2008). Recent studies indicate that disruption of the beclin1-BCL2 complex is an effective mechanism to promote autophagy formation, which prevents premature ageing and improves the health span of mammals (Fernandez et al., 2018). Insufficient autophagy might result in the senescence of pulmonary epithelial cells and the activation of myofibroblasts in pulmonary fibrosis (Araya et al., 2013), and autophagy was inhibited in IPF patients (Patel et al., 2012). In lung epithelial cells, bleomycin could induce pulmonary fibrosis in mice by impeding TFEB-mediated autophagic flux (Wang et al., 2018). AMPK-dependent activation of autophagy enhances collagen turnover to deactivate myofibroblasts, which attenuates BLM-induced pulmonary fibrosis (Rangarajan et al., 2018). mTOR is a key regulator of growth in animals, regulating the balance of apoptosis and autophagy when cells are exposed to physiological stimulation and is also the downstream Wnt signal (Park et al., 2019). Sirolimus, a mTOR (mammalian target of rapamycin) inhibitor, attenuates BLM-induced pulmonary fibrosis in rats (Simler et al., 2002). Therefore, inhibiting Wnt/mTOR signaling could promote autophagy formation, and this is an effective method to attenuate pulmonary fibrosis.

Ellagic acid (EA) is generated by hydrolysis of complex polyphenolic compounds named ellagitannins and is found in a wide variety of fruits and nuts, such black currants, grapes, raspberries, and strawberries (De Ancos et al., 2000; Priyadarsini et al., 2002). Ellagic acid has shown multiple protective effects during fibrotic diseases such as liver fibrosis, pancreatic fibrosis and cardiac fibrosis (Buniatian, 2003; Suzuki et al., 2009; Lin et al., 2019). Ellagic acid promoted apoptosis and autophagy and suppressed Wnt/β-catenin and mTOR pathways in tumour cells (Li et al., 2005; Edderkaoui et al., 2008; Duan et al., 2019). In addition, previous studies have reported that Ellagic acid could attenuate BLM-induced pulmonary fibrosis in rats (Thresiamma and Kuttan, 1997; Saba et al., 2013), but the therapeutic effect of BLM-induced pulmonary fibrosis in mice and its mechanism are not clear. In our studies, we demonstrated that Ellagic acid could alleviate BLM-induced pulmonary fibrosis in mice mainly by inhibiting fibroblast activation and inducing myofibroblast autophagy and apoptosis, and its main mechanism is regulating the Wnt pathway.

## Methods and Materials

### BLM-Induced Animal Model of Pulmonary Fibrosis

Fifty 7- to 8-week-old male C57BL/6J mice were purchased from Charles River (Beijing, China). All mice were housed and cared for in a pathogen-free facility at Nankai University. The mice were acclimatized in a room with constant temperature (25 ± 2°C) and relative humidity (60 ± 2%) and allowed free access to food and water. All animal experiments were approved by the Animal Care and Use Committee (IACUC) at Nankai University (Permit No. SYXK 2014-0003). The mice were randomly divided into five groups (*n* = 10 per group): Control, BLM, Pirfenidone-treated (200 mg/kg), Low-Ellagic acid-treated (10 mg/kg), and High-Ellagic acid-treated (20 mg/kg) groups. Pirfenidone was purchased from Dalian Meilun Biotechnology (Dalian, China), Ellagic acid was purchased from Macklin Biochemical (Shanghai, China). The mice were orally exposed to Ellagic acid, pirfenidone and water once a day for 7–13 days. For BLM administration, mice were anesthetized and then intratracheally injected with bleomycin (Nippon Kayaku Co., Ltd., Tokyo, Japan) at a dose of 2 U/kg body weight for analysis of the fibrotic response. The sham-operated group received intratracheal injections of the same amount of saline. The drug administration group were administered daily from the seventh day of modeling to the 13th day. Mice were sacrificed on day 14. In brief, after anesthetized, the hearts were perfused with PBS through the right ventricle until lungs cleared of blood, and then the right lung tissues were isolated for hydroxyproline assay (three lung lobes) and western blot assay (one lung lobe), the left lung tissues were inflated with 0.5 ml of 10% neutral buffered formalin and went through histology assay.

### The Isolation of Primary Pulmonary Fibroblasts (PPF) and Cell Culture

A mouse lung fibroblast cell line (Mlg), mouse embryonic fibroblast cell line (NIH-3T3) (kindly supplied by Professor Wen Ning, Nankai University) were cultured in DMEM (Solarbio, Beijing, China) supplemented with 10% fetal bovine serum (FBS, Biological Industries, Israel) and antibiotics (100 mg/ml streptomycin and 100 U/ml penicillin G) in a 37°C atmosphere of 95% humidified air and 5% CO_2_. Primary pulmonary fibroblasts (PPFs) were isolated from NaCl- and BLM-treated C57BL/6J mice as mentioned earlier (Ning et al., 2004). Briefly, the lungs were lavaged three times with 1 ml of PBS, digested with 2.5 mg/ml diepase II (Roche, United States), and 2.5 mg/ml collagenase Type 4 (Worthington, United States) for 30 min at 37°C and then centrifuged. The cell pellet was resuspended in DMEM containing 10% FBS and cultured in 5% CO_2_ at 37°C in a humidified atmosphere. PPF cells at passages two to five were used for various assays. Chloroquine (Klionsky et al.) was purchased from Macklin (Shanghai, China), and bafilomycin A1 (Baf A1) was purchased from Cayman Chemical (Wuhan, China).

### Western Blot

Lung tissue and cell samples were homogenized in RIPA lysis (Beyotime Biotechnology, Shanghai, China) buffer with PMSF and NaF (phosphatase inhibitor; added when extracting the phosphorylating protein) and then centrifuged (10,000 rpm, 10 min) to obtain supernatants. The total protein concentration was measured by a BCA Protein Assay kit (Beyotime Biotechnology, Shanghai, China). The secondary antibodies were goat anti-rabbit IgG-HRP (Abcam, Share, United Kingdom) and goat anti-mouse IgG-HRP (Abcam, Share, United Kingdom). The relative density of each band was analyzed by ImageJ. The following primary antibodies were used:

**TABLE 1:** The list of primary antibodies.

Antibody	Company and Item No.	Antibody	Company and Item No.
GAPDH	Affinity, AF7021	P62	Proteintech, 8420-1-AP
β-tubulin	Affinity, T0023	Atg16L1	CST, 8089T
β-catenin	CST, 8480T	LC3A/B	CST, 12741S
p-β-catenin (s33/37/Thr41)	CST, 9561T	mTOR	Abcam, ab32028
p-β-catenin (Thr41/Ser45)	CST, 9565T	p-mTOR (S2448)	Abcam, ab109268
α-SMA	Affinity, BF9212	S6 ribosomal protein (S6RP)	Affinity, AF7831
Collagen I	Affinity, AF7001	p-S6 ribosomal protein (S235/S236)	CST, 4858T
Fibronectin	Affinity, AF5335	Caspase3	CST, 9662S
Akt	SANTA CRUZ, sc-56878	Cleaved-Caspase3	CST, 9664T
p-Akt (Ser473)	SANTA CRUZ, sc-514032	Caspase9	CST, 9009T
Erk1/2	SANTA CRUZ, sc-514302	Cleaved-Caspase9	CST, 9504T
p-Erk1/2 (Thr202/Thy204)	SANTA CRUZ, sc-81492		

### Quantitative Real-Time PCR (qRT-PCR)

Total RNA was extracted using TRIzol Reagent (Invitrogen, Carlsbad, CA, United States). cDNA was obtained from total RNA through reverse transcription. qRT-PCR was performed by using SYBR GreenER qPCR SuperMix Universal (Invitrogen, Carlsbad, CA, United States) according to the manufacturer’s protocols. The relative quantification of gene expression (a-SMA, Collagen I, Fibronectin, Cyclin D1, MMP7, and Wisp1) was measured relative to the endogenous reference gene β-actin using the comparative CT method in the experiment. Sequences of the specific primer sets are as follows:

**TABLE 2:** The list of gene primers.

Gene	Forward primer sequence	Reverse primer sequence
α-SMA (NM_007392.2)	GCT​GGT​GAT​GAT​GCT​CCC​A	GCC​CAT​TCC​AAC​CAT​TAC​TCC
Fn (NM_010233.1)	GTG​TAG​CAC​AAC​TTC​CAA​TTA​CGA​A	GGA​ATT​TCC​GCC​TCG​AGT​CT
Col1 a1 (NM_007742.3)	CCA​AGA​AGA​CAT​CCC​TGA​AGT​CA	TGCACGTCATCGCACACA
Cyclin D1 (NM_007631)	GCG​TAC​CCT​GAC​ACC​AAT​CT	CAG​GTC​TCC​TCC​GTC​TTG​AG
Wisp1 (NM_018865)	CAG​CAC​CAC​TAG​AGG​AAA​CGA	CTG​GGC​ACA​TAT​CTT​ACA​GCA​TT
β-actin (NM_007393.3)	AGG​CCA​ACC​GTG​AAA​AGA​TG	AGA​GCA​TAG​CCC​TCG​TAG​ATG​G

### Hematoxylin-Eosin (H and E) and Masson Staining

Left lungs were fixed in 10% formalin for 24 h and embedded in paraffin. Then, lung sections (5 µm) were prepared and stained with hematoxylin-eosin or Masson’s trichrome with commercial kits according to the manufacturers’ instructions (Solarbio, Beijing, China). The digitized images of the slides were collected using an upright microscope (Leica DM1000, Germany). Quantification of pulmonary fibrosis was performed as described previously (Jiang et al., 2004). In brief, Masson staining slides were examined under high power (×10 objective), and five random fields per specimen were captured. Then, digitized images were opened in Image-Pro Plus Version 6.0 (Media Cybernetics, Inc., United States), and the software selection tool was used to select the entire lung tissue area and automatically calculate the total pixel (Pw) of the region. Then, the same method was used to calculate the total pixel (Pf) of the fibrosis region, fibrosis ratio = fibrosis area pixel (Pf)/total lung pixel (Pw).

### GFP-LC3 and Cherry-GFP-LC3 Transfections and Immunofluorescence

GFP-LC3B (mouse) and Cherry-GFP-LC3B (mouse) plasmids were transfected into NIH-3T3 cells using PEI according to the supplier’s protocol (Sino Biological Inc., Beijing, China). Cells were fixed in 4% paraformaldehyde for 20 min, washed with PBS, permeabilized with 0.2% Triton X-100 in PBS, blocked with 5% BSA and incubated with α-SMA antibodies. Cells were washed with PBS, and FITC was used for immunofluorescence visualization. Next, tissues were incubated with anti- α-SMA and anti-Col1 antibodies overnight at 4°C and then with secondary FITC- or rhodamine (TRITC)-labelled antibodies for 30 min. Fluorescein (FITC)-conjugated AffiniPure goat anti-mouse IgG (H + L) and rhodamine (TRITC)-conjugated Affinipure goat anti-rabbit IgG (H + L) were purchased from Jackson ImmunoResearch (Pennsylvania, Unites States). Nuclei were stained with DAPI (Solarbio, Beijing, China), and cells and tissues were photographed with a TCS SP8 confocal microscope (Leica).

### Dual Luciferase Assay

TCF/LEF promoters were cloned into the pGL4.49 luciferase reporter vector, and NIH-3T3 cells were transfected with luciferase reporter plasmids using PEI. Renilla-luciferase was used as an internal control. Cells were treated 18 h after transfection with a series of EAs for 8 h. Cells were harvested, and the luciferase activity of cell lysates was determined using a luciferase assay system (Promega, United States) as described by the manufacturer. Total light emission was measured using a GloMax®-Multi Detection System (Promega, United States).

### Immunohistochemistry

The tissue sections were pre-treated in a microwave, blocked and incubated using a series of antibodies, and stained with DAB and hematoxylin. The results were captured using a microscope (Nikon, Japan). The method of counting the positive area is as follows: 1) Using ImageJ to open a picture, click “Image” and “type”, change “RGB Color” to “RGB stack”; 2) Click “Image”, drop down and click “adjust”, change the B and W in the “Default” column to “Red”; 3) adjust the upper and lower pulleys to select the positive signal area, click “set”, click “ok”; 4) Then, click “analyze”, click “set measurement”, choose “area fraction”; 5) Begin to count positive results, click “Control + M” on the keyboard, the number of “the% Aera” is the result.

### Flow Cytometric Analysis of Apoptosis

NaCl-PPF or PPF-BLM cells/ml (1 × 10^6^) were seeded into six-well plates and left for 24 h in an incubator to resume exponential growth. The extent of apoptosis was measured using an annexinV-FITC apoptosis detection kit (Beyotime, Shanghai, China) as described by the manufacturer’s instructions. Cells were exposed to the drug and incubated for 24 h. Then, they were collected and washed with PBS twice, and gently resuspended in annexin-V binding buffer and incubated with annexinV-FITC/PI in the dark for 15 min. The number of apoptotic cells was detected by CytoFLEX S (Beckman Coulter, United States).

### Hydroxyproline Assay

The collagen contents in the right lungs of mice were measured with a conventional hydroxyproline method (Taylor et al., 2002). In brief, the right lungs were dried and acid-hydrolyzed, the residue was filtered, and the pH value was adjusted to 6.5–8.0. Hydroxyproline analysis was performed using chloramine-T spectrophotometric absorbance as previously described.

### Evaluation of Pulmonary Function

C57BL/6J mice were administered BLM (2 U/kg) on day 0; orally exposed to Ellagic acid, pirfenidone and water once a day for days 7–13; and finally, sacrificed on day 14. After the mice were anesthetized, we carefully cut the neck skin with a scalpel (trying not to bleed, otherwise it would affect the experimental data of lung functions) and subsequently used the surgical line to fix the cannula. The mice were transferred into the plethysmography chamber, and functions were analyzed using the Anires2005 system (Biolab, Beijing, China). This system automatically calculates and displays pulmonary function parameters, including forced vital capacity (FVC), dynamic compliance (Cydn), inspiratory resistance (Ri) and expiratory resistance (Re).

### Statistical Analysis

Statistical analyses was performed using GraphPad Prism 6.0 software. One statistical approach that was One-way ANOVA with Greenhouse-Geisser correction followed by Dunnett’s multiple comparisons test was used to calculate the significance of the differences between group means, and all data expressed as mean ± (Standard Error of Mean) SEM, *p < 0.05, **p < 0.01, ***p < 0.001, and NS: nonsignificant (one-way ANOVA).

## Results

### Ellagic Acid Attenuates BLM-Induced Pulmonary Fibrosis in Mice

**FIGURE 1:**
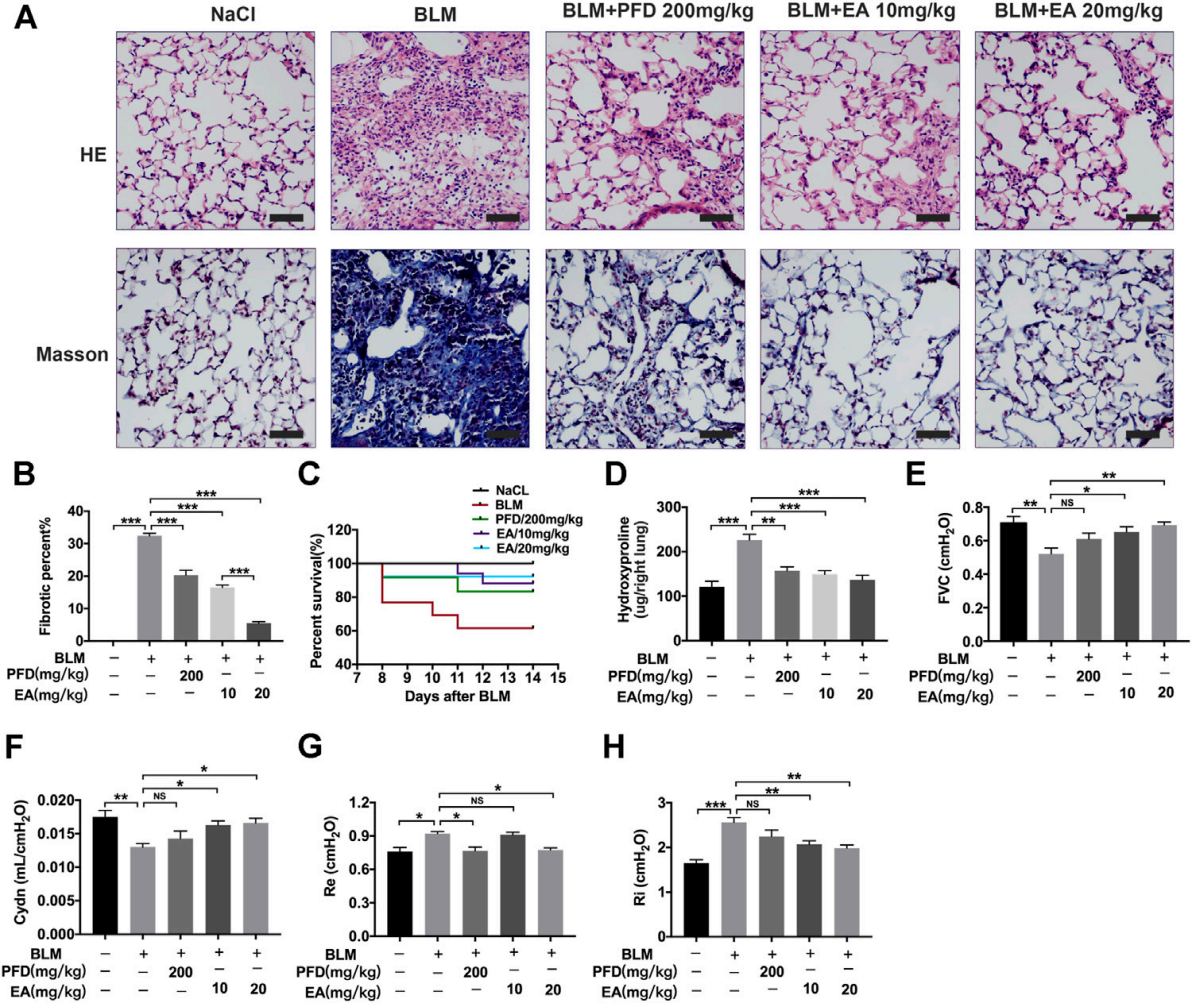
Ellagic acid attenuates BLM-induced pulmonary fibrosis in mice. Ellagic acid (10 mg/kg, 20 mg/kg) and pirfenidone (200 mg/kg) were given orally once a day from day 7–13 after BLM treatment and lungs were harvested on day 14 **(A)** Representative images of hematoxylin-eosin (H and E) and Masson staining of lung tissue sections. Scale bars: 50 μm **(B)** Percentages of fibrotic area in each group **(C)** Percentages of surviving mice were plotted from day 7–13 after BLM treatment **(D)** Hydroxyproline contents in right lung tissues **(E–H)** Parameters of lung function such as forced vital capacity (FVC), dynamic compliance (Cydn), expiratory resistance (Re) and inspiratory resistance (Ri). Data (Figures 1B–H) are means ± Standard Error of Mean, *n* = 6, *p < 0.05, **p < 0.01, ***p < 0.001, and NS: nonsignificant (one-way ANOVA).

To determine the therapeutic impact of Ellagic acid on pulmonary fibrosis in mice, a BLM-induced pulmonary fibrosis mouse model was established, and mice were treated with Ellagic acid after exposure to BLM. Pirfenidone is one of the listed drugs for the treatment of IPF and was used as a positive drug. We treated C57BL/6 J mice with Ellagic acid (10 mg/kg, 20 mg/kg) from day 7 to day 13 after administrating BLM, and mice were sacrificed on day 14. Hematoxylin-eosin and Masson staining results indicated that Ellagic acid improved alveolar structure distortion and decreased the fibrotic percent (Figures 1A,B). The dead ratio of mice sharply increased and reached up to 60% when the mice were exposed to BLM, and Ellagic acid was able to increase the survival percentage of mice (Figure 1C). In addition, in the right lung tissue, Ellagic acid reduced the level of hydroxyproline (Figure 1D). Lung functions are a key indicator of treatment efficiency in clinical trials. Ellagic acid has positive impacts on lung functions such as forced vital capacity (FVC), dynamic compliance (Cydn), expiratory resistance (Re), and inspiratory resistance (Ri) (Figures 1E–H). These data indicate that Ellagic acid effectively attenuated BLM-induced pulmonary fibrosis in mice and that the therapeutic effect of Ellagic acid was better than that of the positive drug pirfenidone.

### Ellagic Acid Inhibits the Wnt Signaling Pathway

**FIGURE 2:**
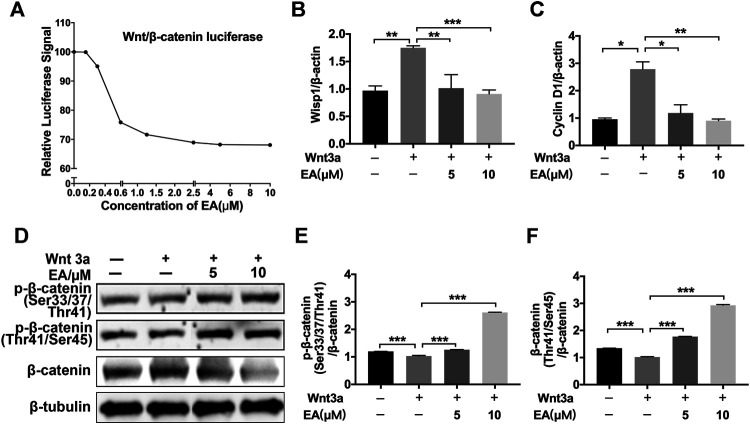
Ellagic acid inhibits Wnt/β-catenin signaling pathway **(A)** NIH-3T3 cells were transfected with the TOPFlash plasmids. After 18 h of transfection, the cells were treated with Wnt3a (100 ng/ml) and/or Ellagic acid at various concentrations for 8 h and then lyzed for luciferase assays **(B**,**C)** NaCl-PPF cells were incubated with Ellagic acid (5 µM, 10 µM) and/or Wnt3a (100 ng/ml) for 12 h to analyze the mRNA levels of Wisp1 and Cyclin D1 by using quantitative real-time PCR **(D)** NaCl-PPF cells were treated with Ellagic acid (5 µM, 10 µM) and/or Wnt3a (100 ng/ml) for 4 h, p-β-catenin (Ser33/37/Thr41) and p-β-catenin (Thr41/Ser45) were detected by Western blot. Densitometric analyses were shown beside. Data (Figures 2B–F) are means ± Standard Error of Mean, *n* = 3, *p < 0.05, **p < 0.01, ***p < 0.001, and NS: nonsignificant (one-way ANOVA). β-tubulin was used as a loading control.

Ellagic acid could attenuate Wnt signaling in other disease models, such as brain injury, HBP carcinomas and skin photoaging (Anitha et al., 2013; Liu et al., 2017; Moon et al., 2018), but whether Ellagic acid inhibits Wnt signaling in pulmonary fibrosis has not been definitely proven. Hence, we explored the effect of Ellagic acid on canonical Wnt signaling in pulmonary fibroblasts. We tested the ability of Ellagic acid to inhibit β-catenin/TCF reporter activity, which was measured with reporter genes harboring TCF/LEF-binding sites. Ellagic acid dose-dependently inhibited Wnt3a-induced TOPFlash activity in NIH-3T3 cells (Figure 2A) and inhibited the expression of Wnt signaling target genes, including CyclinD1 and Wisp1 in NaCl-PPF cells (Figures 2B,C). Further investigations indicated that Ellagic acid increased the ratio of phospho-β-catenin (Ser33/37/Thr41 and Thr41/Ser45) to β-catenin (Figures 2D–F). *In vivo* experiments also indicated that Wnt3a and β-catenin were overexpressed in lung tissues at day 7 and day 14 after BLM treatment and Ellagic acid inhibited the overexpression of Wnt3a and β-catenin (Supplementary Figure S1). We further tested the expression of β-catenin in BLM-PPF cells and the result was consistent with that of *in vivo* (Supplementary Figure S2). Therefore, these data demonstrate that Ellagic acid suppresses the canonical Wnt signaling pathway.

### Ellagic Acid Suppresses Wnt3a-Induced Fibroblast Activation and ECM Accumulation by Regulating the Phosphorylation of Erk and Akt

**FIGURE 3:**
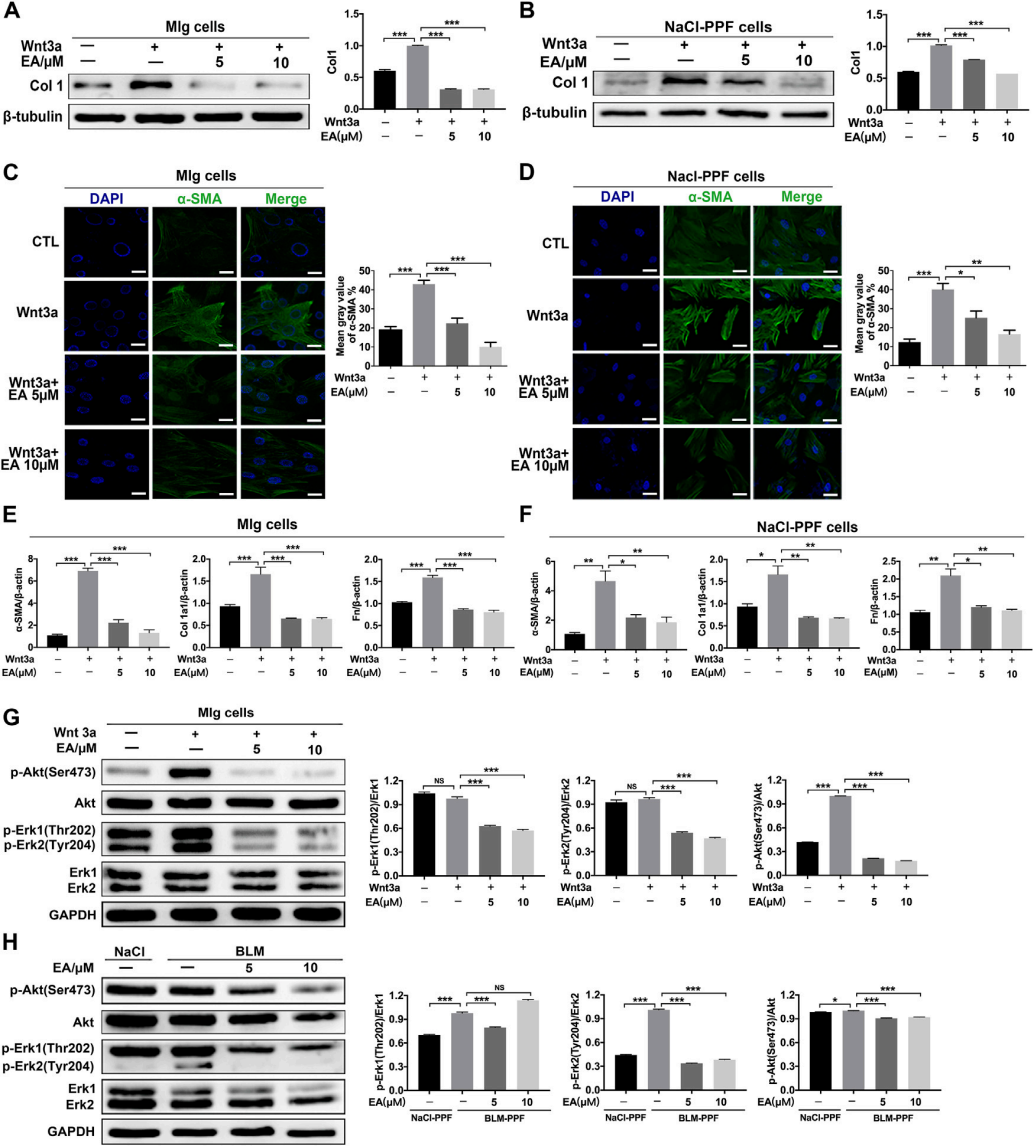
Ellagic acid decreases Wnt3a-induced pulmonary fibroblasts activation and ECM accumulation **(A**,**B)** Mlg and NaCl-PPF cells were exposed to Wnt3a (100 ng/ml) and/or Ellagic acid (5 µM, 10 µM) 24 h to detect expression level of Col1 by using Western blot. Densitometric analyses were shown beside **(C**,**D)** Mlg and NaCl-PPF cells were exposed to Wnt3a (100 ng/ml) and/or Ellagic acid (5 µM, 10 µM) 24 h to detect the α-SMA expression level by immunofluorescence, the analyses of mean gray value were shown beside **(E**,**F)** Wnt3a (100 ng/ml) and/or Ellagic acid (5 µM, 10 µM) were incubated with Mlg and NaCl-PPF cells for 12 h, and cell lysate was used to analyze the mRNA levels of α-SMA, Col1 and Fn by using quantitative real-time PCR(*n* = 3) **(G**,**H)** Mlg and NaCl-PPF cells were exposed to Wnt3a (100 ng/ml) and/or Ellagic acid (5 µM, 10 µM) for 12 h, BLM-PPF cells were treated with Ellagic acid (5 µM, 10 µM) for 12 h to evaluate protein expression levels of p-Akt (Ser473), Akt, p-Erk1/2 (Thr202/Tyr204)and Erk1/2 by western blot. Densitometric analyses were shown beside. Data (Figures 4A–H) are means ± Standard Error of Mean, *n* = 3, *p < 0.05, **p < 0.01, ***p < 0.001, and NS: nonsignificant (one-way ANOVA). β-tubulin or GAPDH was used as a loading control.

We further explored whether Ellagic acid could decrease Wnt3a-induced myofibroblast activation and ECM production and its underlying mechanism. The results indicate that Ellagic acid could significantly decrease Wnt3a-induced Col 1 expression in Mlg cells and NaCl-PPF cells (Figures 3A,B). The immunofluorescence results also revealed that Ellagic acid suppressed Wnt3a-induced expression of α-SMA in Mlg and NaCl-PPF cells (Figures 3C,D). Ellagic acid also downregulated Wnt3a-induced mRNA levels of α-SMA, Col1 a1 and Fn in Mlg and NaCl-PPF cells (Figures 3E,F). To further explore the mechanism, we detected whether Ellagic acid could affect downstream Wnt signaling. Since Wnt3a activates the proliferation of fibroblast cells via activation of both the Erk and Akt pathways (Yun et al., 2005; Kim et al., 2007), we further confirmed that Ellagic acid significantly decreased the Wnt3a-induced proportions of p-Erk1 (Thr202)/Erk1, p-Erk2 (Tyr204)/Erk2, and p-Akt (Ser473)/Akt in Mlg cells (Figure 3G), and BLM-PPF (BLM-primary pulmonary fibroblasts) cells incubated with Ellagic acid also showed similar results (Figure 3H). These results show that Ellagic could significantly decrease Wnt3a-induced pulmonary fibroblast activation and ECM production by inhibiting the activation of Erk1 (Thr202), Erk2 (Tyr204), and Akt (Ser475) in pulmonary fibroblasts.

### Ellagic Promotes Pulmonary Fibroblast Autophagy by Inhibiting the Wnt-mTOR Signaling Pathway

**FIGURE 4:**
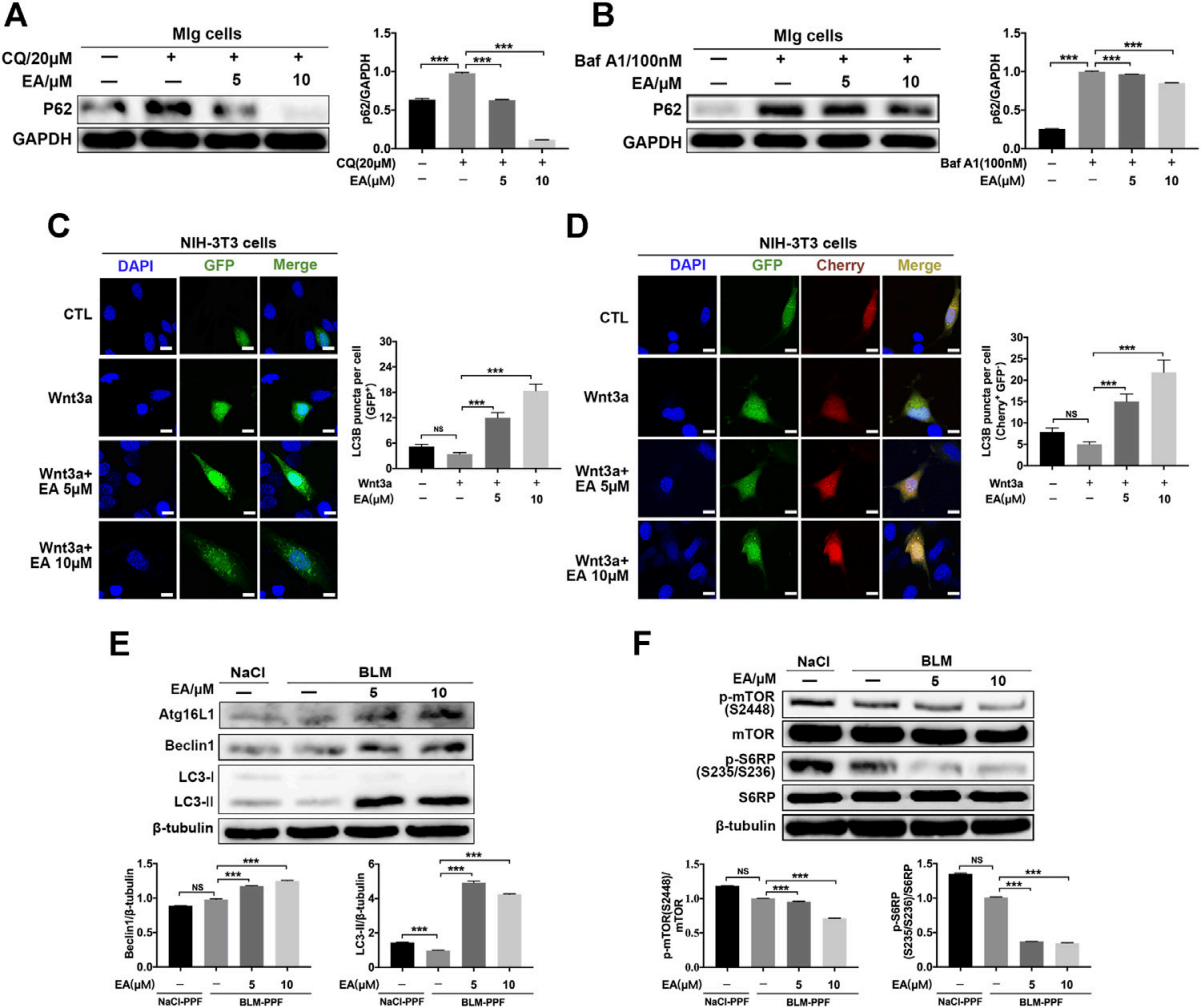
Ellagic acid promotes pulmonary fibroblast autophagy mainly via inhibiting Wnt-mTOR signaling pathway **(A**,**B)** Mlg cells were exposed to CQ (20 µM) and Baf A1 (100 nM) with or without Ellagic acid (5 µM, 10 µM) to analyze the p62 expression level by using western blot. Densitometric analyses were shown beside **(C**,**D)** The plasmids of GFP-LC3B and mCherry-GFP-LC3B were transfected to NIH3T3 cells with PEI, and these cells were subsequently exposed to Ellagic acid (5 µM, 10 µM) and/or Wnt3a (100 ng/ml) for 12 h. DNA was counterstained with DAPI (blue). Quantitative analyses are showed beside. Scale bars: 50 μm **(E)** BLM-PPF cells were treated with Ellagic acid (5 µM, 10 µM) for 24 h, and the Atg16L1, Beclin1 and LC3-II/I expression levels were detected by Western blot. Densitometric analyses were shown below **(F)** BLM-PPF cells were treated with Ellagic acid (5 µM, 10 µM) for 12 h, and protein expression levels of mTOR, S6RP and their phosphorylation were detected by Western blot. Densitometric analyses were shown below. Data are means ± Standard Error of Mean, *n* = 3, *p < 0.05, **p < 0.01, ***p < 0.001, and NS: nonsignificant (one-way ANOVA). β-tubulin or GAPDH were used as a loading control.

The Wnt pathway is a well-known pathway and involves the regulation of autophagy (Ma et al., 2005; Semenov et al., 2007; Saiki et al., 2011). To explore whether Ellagic acid impacts autophagy and downstream Wnt signaling (mTOR signaling), we established a cellular model of inhibitory autophagy. p62 protein accumulation suggests that autophagy activity was decreased (Katsuragi et al., 2015). The two autophagy inhibitors, Chloroquine (CQ) and Bafilomycin a1 (Baf a1), could increase the expression levels of p62 (Supplementary Figure S3), and Ellagic acid significantly downregulated CQ and Baf A1-induced p62 protein expression in Mlg cells (Figures 4A,B). GFP-LC3B and mCherry-GFP-LC3B plasmids were used to detect autophagic flux. LC3B, its N terminus with a fluorescent protein such as GFP (GFP-LC3B), has been used to monitor autophagy through fluorescence microscopy, and the number of green puncta in cells indicated the quantity of GFP-LC3B puncta (Klionsky et al., 2016). A method that is designed to detect autophagy flux on the use of a tandem monomeric mCherry-GFP-tagged LC3B, and the number of green puncta and yellow puncta after merging mCherry and GFP indicated the quantity of autophagosomes and autolysosomes, respectively, (Klionsky et al., 2016). Our results showed that Ellagic acid increased Wnt3a-induced GFP-LC3B puncta in NIH-3T3 cells (Figure 4C). To corroborate these findings, we used mCherry-GFP-LC3B reporters to measure the formation of autophagosomes (Cherry^+^ GFP^+^ signal) and autolysosomes (Cherry^+^ GFP^–^signal) in NIH3T3 cells. As expected, Ellagic acid treatment significantly increased Wnt3a-induced Cherry^+^ GFP^–^puncta (autolysosomes) (Figure 4D). In addition, we found that Ellagic acid promoted the expression of autophagy-related proteins such as Atg16 L, Beclin1, and LC3-II (LC3 lipidation) (Figure 4E). The mTOR pathway is the downstream Wnt signaling pathway and is related to autophagy formation, so we examined the protein expression of mTOR and S6RP (S6 ribosomal protein) and their phosphorylation by Western blot. Our studies revealed that the administration of Ellagic acid reduced phosphorylation levels of mTOR (S2248) and S6RP (S235/S236) in BLM-PPF cells (Figure 4F). Therefore, these data suggest that Ellagic acid induced autophagy formation by suppressing the Wnt-mTOR signaling pathway in pulmonary fibroblasts.

### Ellagic Acid Promotes the Apoptosis of Pulmonary Myofibroblasts

**FIGURE 5:**
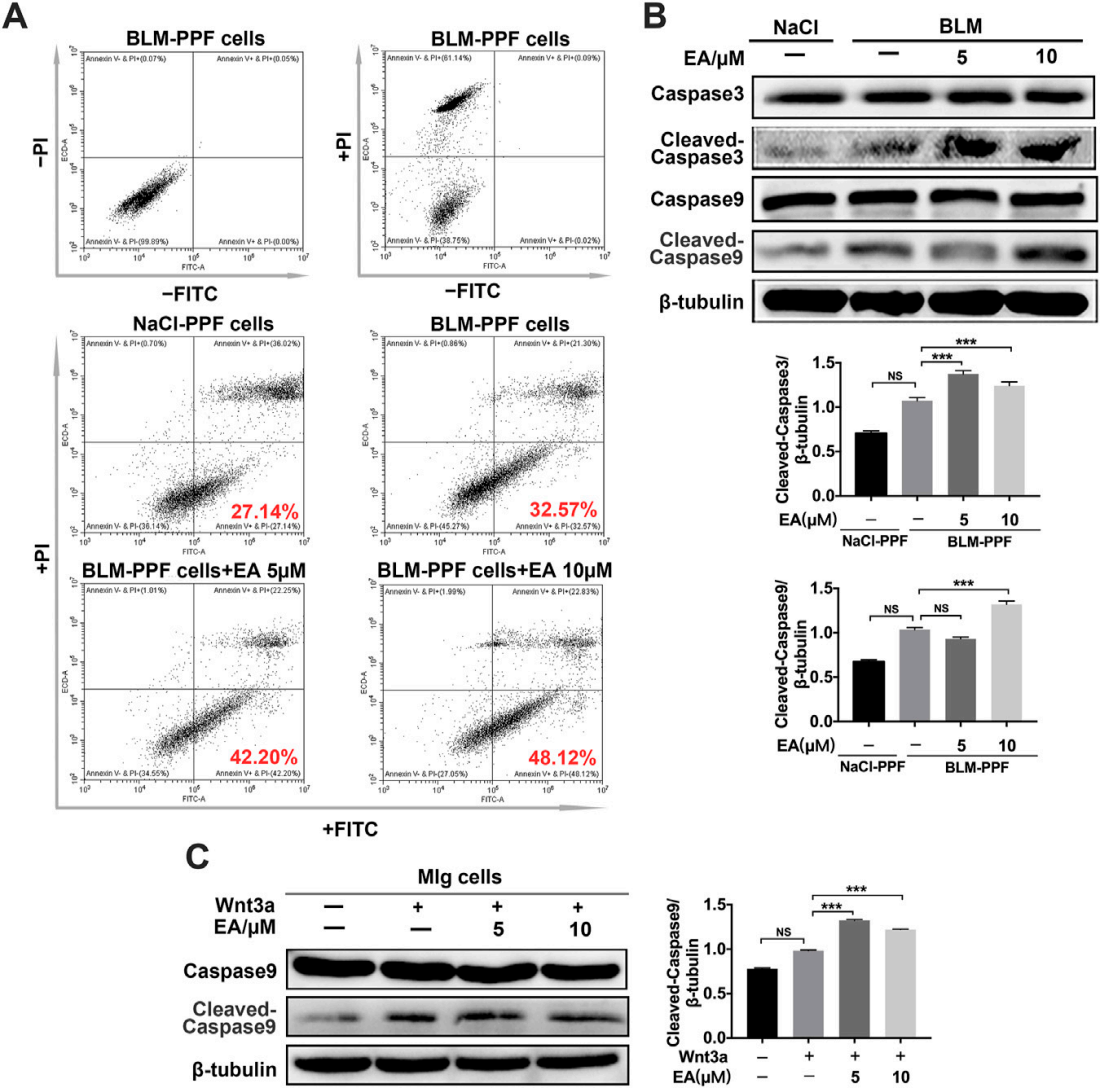
Ellagic acid promotes the apoptosis of pulmonary myofibroblasts **(A)** BLM-PPF cells incubated with Ellagic acid (5 µM, 10 µM) and/or Wnt3a (100 ng/ml) for 24 h, and Annexin V/PI staining was subsequently performed to estimate early, late apoptosis and necrosis by flow cytometry **(B)** BLM-PPF cells were treated with Ellagic acid (5 µM, 10 µM) for 24 h, Lysates were immunoblotted for Caspase3, Cleaved-Caspase3, Caspase9 and Cleaved-Caspase9. Densitometric analyses were shown below **(C)** Mlg cells were treated with Ellagic acid (5 µM, 10 µM) and/or Wnt3a (100 ng/ml) for 24 h, Lysates were immunoblotted for Caspase9 and Cleaved-Caspase9. Densitometric analysis were shown beside. Data in **(B**,**C)** are means ± Standard Error of Mean, *n* = 3, ***p < 0.001, and NS: nonsignificant (one-way ANOVA). β-tubulin was used as a loading control.

To detect whether Ellagic acid contributed to apoptosis*,* we analyzed the number of apoptotic cells using flow cytometry. BLM-PPF cells were exposed to Ellagic acid to analyze the number of apoptosis cells by using flow cytometry, and double negative (not add PI and Annex-V-FITC staining) and single negative (add PI and not add Annex-V-FITC staining) were important tests to draw the gate. Treatment with Ellagic acid resulted in an increase in annexin V^+^/PI^−^cells (early apoptosis) but no change in Annexin V^+^/PI^+^ (late apoptosis) or annexin V^−^/PI^+^ cells (necrosis) (Figure 5A). In addition, caspase proteins are tightly related to cellular apoptosis. Our experiments indicated that Ellagic acid increased the expression of cleaved caspase-9 and cleaved caspase-3 in PPF cells (Figure 5B) and Wnt3a-induced the expression of cleaved caspase-9 in Mlg cells (Figure 5C). Hence, Ellagic acid could promote the apoptosis of pulmonary myofibroblasts.

### Ellagic Attenuates BLM-Induced Fibroblast Activation and ECM Accumulation *in vivo*

**FIGURE 6:**
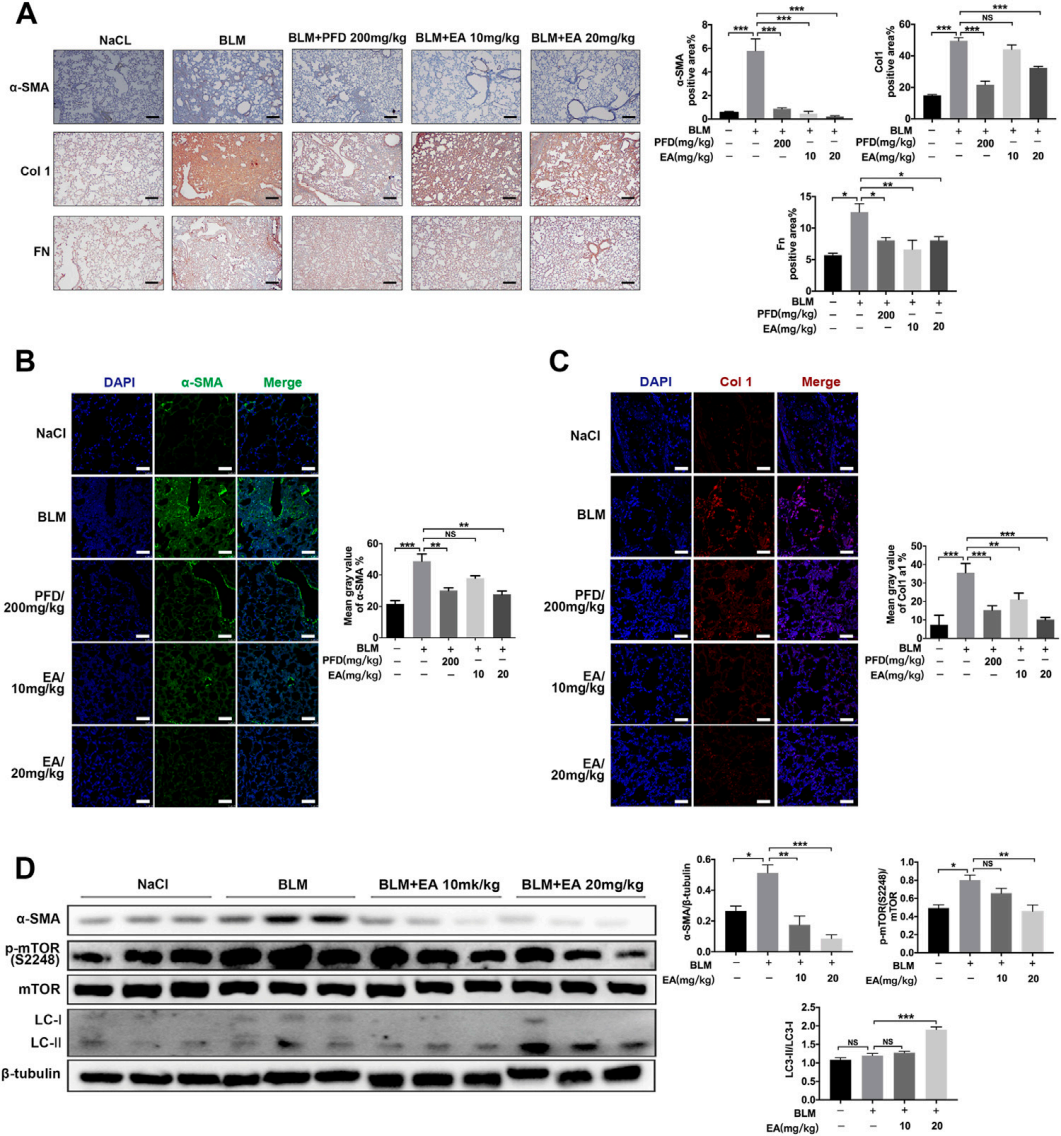
Ellagic acid attenuates BLM-induced fibroblasts activation and ECM accumulation *in vivo*. The mice were treated with Ellagic acid (10 mg/kg, 20 mg/kg) from day 7–13 after administrating BLM **(A)** Immunohistochemistry was used to analyze the expression levels of α-SMA, Col1 and Fn (*n* = 3). Quantitative analysis was shown beside. Scale bars: 50 μm **(B**–**C)** The expression levels of α-SMA and Col1 were detected by immunofluorescence in lung sections. The analyses of mean gray value were shown beside. Scale bars: 50 μm **(D)** Lung homogenization was used to analysis the α-SMA, LC3-II/I and *p*-mTOR (S2248), mTOR expression levels by Western blot (*n* = 6). Densitometric analyses were shown beside. Data in **(A**,**D)** are means ± Standard Error of Mean, *p < 0.05, **p < 0.01, ***p < 0.001, and NS: nonsignificant (one-way ANOVA). β-tubulin was used as a loading control.

The mice were treated with Ellagic acid on day 7 to day 13, and we employed immunohistochemistry to identify that Ellagic acid had a negative impact on the expression of α-SMA, Col 1 and Fn in lung sections, which were equal to the results of the positive drug pirfenidone (Figure 6A). Our immunofluorescence results also showed inhibitory expression of α-SMA and Col1 in lung sections (Figures 6B,C). In addition, we used a tissue homogenizer to lyse lung tissues, and α-SMA, LC3-1Ⅰ/Ⅱ, p-mTOR, and mTOR protein levels were analyzed by using Western blotting. Ellagic acid decreased α-SMA expression and the ratio of p-mTOR/mTOR and increased the ratio of LC3-II/LC3-I (Figure 6D). Together, these data identified that Ellagic acid significantly inhibited myofibroblast activation and mTOR signaling and promoted autophagy formation *in vivo.*

## Discussion

Idiopathic pulmonary fibrosis is a chronic, fatal lung disease, and many IPF patients experience respiratory failure (Mora et al., 2017). The importance of the Wnt signaling pathway has been identified in pulmonary fibrosis, and the main reason is that aberrant Wnt signaling could induce fibroblast activation and ECM production (Morrisey, 2003; Baarsma et al., 2011). When Wnt signaling is abnormally activated, β-catenin is translocated into the nucleus to upregulate related target genes such as Cyclin D1, MMP7, and Wisp1 (Crawford et al., 1999; Konigshoff et al., 2009; Zhang et al., 2012). In our studies, Ellagic acid inhibited the Wnt/β-catenin signaling pathway by decreasing the expression of Cyclin D1, MMP7, Wisp1, and nuclear β-catenin and increasing the phosphorylation of β-catenin.

Collagen, a main component of extracellular protein, maintains the basic structure of the lung. However, there are overactivated fibroblasts and overexpressed ECM proteins in fibroblastic foci, and inhibiting this condition could effectively attenuate pulmonary fibrosis (Snijder et al., 2019). Excessive ECM proteins result in the formation of lung scars, which might accelerate the decline in force vital capacity (Bates et al., 2007; King et al., 2011). Previous studies showed that Wnt ligands promoted fibroblast activation and collagen synthesis (Konigshoff et al., 2008) and increased the phosphorylation levels of Akt and Erk (Yun et al., 2005; Kim et al., 2007). Therefore, there is an effective method to inhibit fibroblast activation and ECM accumulation by downregulating Wnt3a-induced activation of Akt and Erk. Our experimental results revealed that Ellagic acid significantly inhibited fibroblast activation and ECM production by decreasing the translation and transcription levels of α-SMA, Col 1 and Fn *in vivo or in vitro*, and the underlying mechanism was mainly the inhibitory Wnt/Akt and Erk signaling.

Insufficient autophagy plays an essential role in the pathogenesis of IPF. Many researchers focus on fibroblast activation because selected autophagy could promote collagen turnover (Rangarajan et al., 2018). The PB1 domain of the p62 protein is involved in the degradation of polyubiquitinated, misfolded, aggregated proteins and dysfunctional organelles by regulating autophagy formation in mammalian cells (Moscat and Diaz-Meco, 2009). Our studies showed that Ellagic acid could increase the expression of p62 in pulmonary fibroblasts. LC3-II, a processed form of LC3, is localized in the membrane of autophagosomes and is regarded as a powerful marker for autophagosomes (Kabeya et al., 2000). During the formation of early autophagosomes, some complexes formed by LC3-II and other autophagy-related proteins are required (Tanida et al., 2004). In addition, Atg16 L^1^ and beclin1 are essential factors in autophagic flux and its biological function (Matsushita et al., 2007; Wirawan et al., 2012). In BLM primary pulmonary fibroblasts, Ellagic acid treatment increased LC3-II, Atg16 L^1^ and beclin1 expression. The mTOR pathway also plays an important role in regulating balanced growth and autophagy in response to environmental stress, and this signal is regulated by Wnt signaling (Yang and Guan, 2007). Interestingly, our results showed that Ellagic acid significantly inhibited Wnt3a-induced activation of mTOR signaling in pulmonary fibroblasts. Hence, Ellagic acid could induce autophagy, and this process might be regulated by the Wnt/mTOR signaling pathway in pulmonary fibrosis. Inhibition of autophagy (by knockout of Atg3, Atg5, Atg7 and mutation of Atg1, Atg13, vsp34) suppresses the induction of apoptosis (Nezis et al., 2010; Rubinstein et al., 2011; Young et al., 2012). Autophagy can degrade catalase and promote necrotic cell death (Yu et al., 2006). In chronic lymphocytic leukaemia cells, downregulating the Wnt signaling pathway promoted cell apoptosis (Lu et al., 2011). Ellagic acid induced apoptosis by increasing the number of early apoptotic cells and promoting the activation of Caspase3 and Caspase9 in Mlg and PPF cells.

In conclusion, we identified that Ellagic acid alleviated BLM-induced pulmonary function and improved lung function in mice. *In vitro* experiments revealed that Ellagic acid could inhibit fibroblast activation and ECM production and promote myofibroblast autophagy and apoptosis by downregulating the Wnt signaling pathway. Ellagic acid also inhibited myofibroblast activation and promoted autophagy formation *in vivo*. Therefore, our studies support for the use of Ellagic acid as a candidate compound for anti-pulmonary fibrosis drugs and provide more potential therapeutic options for IPF patients.

## Data Availability

The original contributions presented in the study are included in the article/Supplementary Material, further inquiries can be directed to the corresponding authors.

## Abbreviations

BLM, Bleomycin; IPF, Idiopathic pulmonary fibrosis; ECM, Extracellular matrix; EA, Ellagic acid; mTOR, mammalian target of rapamycin; BCL2, B-cell lymphoma-2; TFEB, Transcription factor EB; PPF, Primary pulmonary fibroblasts; Mlg, Mouse lung fibroblasts cell; DMEM, dulbecco’s modified eagle medium; NIH-3T3, Mouse embryonic fibroblasts cell line; FBS, Fetal bovine serum; CQ, Chloroquine; Baf A1, Bafilomycin A1; PMSF, Phenylmethylsulfonyl fluoride; PEI, Polyethylenimine; FVC, forced vital capacity; Cydn, dynamic compliance; Ri, inspiratory resistance; Re, expiratory resistance; Pf, fibrosis area pixel; Pw, total lung pixel; H-E, Hematoxylin-eosin; TGF-β, Transforming growth factor β; Fn, Fibronectin; Col 1, Collagen I; α-SMA, α-smooth muscle actin; LC3, Microtubules associated protein 1 light chain 3; MMP, Matrix metalloproteinase.
