# Supplementary material for: Ellagic Acid Attenuates BLM-Induced Pulmonary Fibrosis via Inhibiting Wnt Signaling Pathway
Source: Front Pharmacol. 2021 Apr 12;12:639574. doi: 10.3389/fphar.2021.639574 (PMC8072668; doi:10.3389/fphar.2021.639574)
Supplement: Supplementary file 1 [file datasheet1.docx]

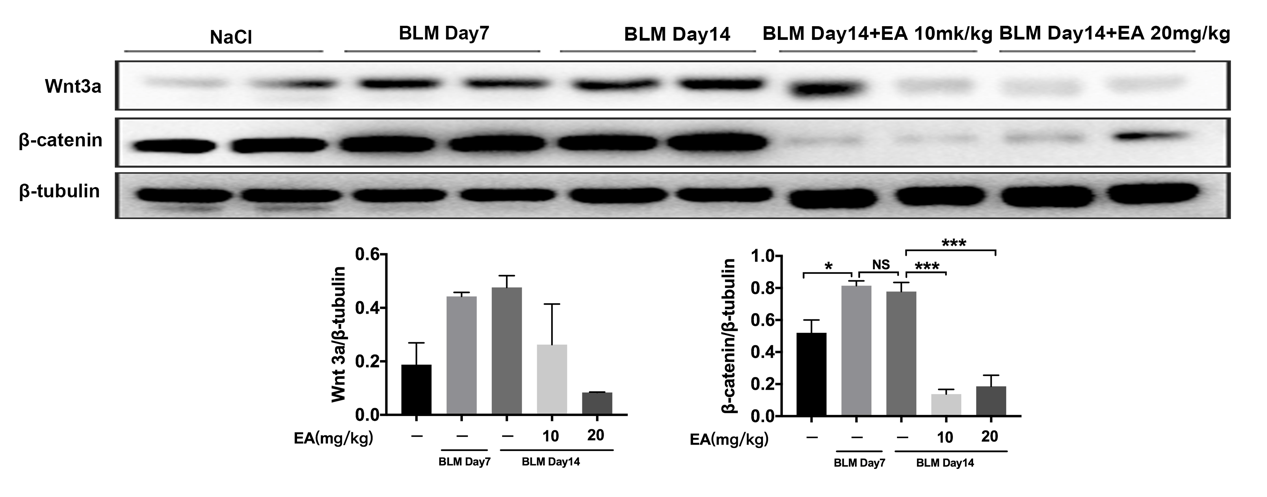


**Fig.S1 Ellgaic acid inhibits Wnt/β-catenin signal *in vivo****.* The mice were treated with Ellagic acid (10mg/kg, 20mg/kg) from day 7-13 after administrating BLM, lung homogenization was used to analysis the Wnt3a and β-catenin expression levels by Western blot. Densitometric analyses were shown below. Data in Fig.S1 are means ±Standard Error of Mean (SEM), n=2, *P < 0.05, ***P < 0.001, NS: nonsignificant (one-way ANOVA). β-tubulin was used as a loading control.


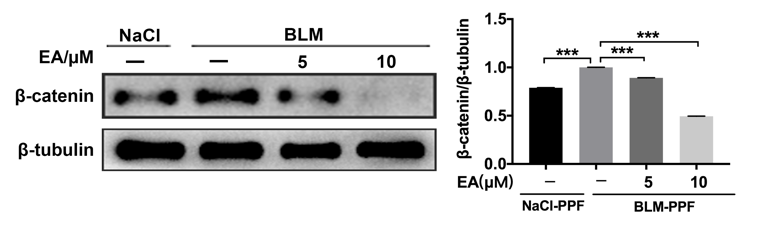


**Fig.S2 Ellgaic acid suppresses the protein expression of β-catenin in BLM-PPF cells.** BLM-PPF cells were treated with Ellagic acid (5µM, 10µM) for 24 hours, and the β-catenin expression level was detected by Western blot. Densitometric analyses were shown beside. Data in Fig.S2 are means ±Standard Error of Mean (SEM), n=3, ***P < 0.001 (one-way ANOVA). β-tubulin was used as a loading control.


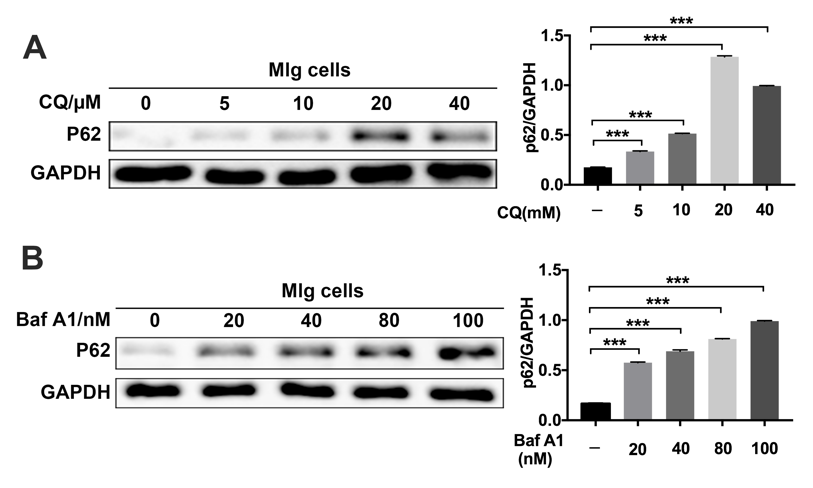


**Fig.S3 Chloroquine (CQ) and Bafilomycin A1(Baf A1) promotes the p62 protein expression in Mlg cells.** (A) Mlg cells were exposed to CQ in a serious concentration for 24 hours. (B) Mlg cells were exposed to Baf A1 in a serious concentration for 24 hours, analyzing the p62 expression level by using western blot. Densitometric analyses were shown beside. Data in Fig.S3A-B are means ±Standard Error of Mean (SEM), n=3, ***P < 0.001 (one-way ANOVA). GAPDH was used as a loading control.
